# A qualitative study of cognitive behavioural therapy in multiple sclerosis: experiences of psychotherapists

**DOI:** 10.1080/17482631.2017.1325673

**Published:** 2017-05-25

**Authors:** Charlotte Ytterberg, Charlotte Chruzander, Gunnel Backenroth, Marie Kierkegaard, Gerd Ahlström, Kristina Gottberg

**Affiliations:** ^a^ Department of Neurobiology, Care Sciences and Society, Karolinska Institutet, Huddinge, Sweden; ^b^ Functional Area Occupational Therapy & Physiotherapy, Karolinska University Hospital, Stockholm, Sweden; ^c^ Department of Clinical Neuroscience, Karolinska Institutet, Stockholm, Sweden; ^d^ Department of Health Sciences, Faculty of Medicine, Lund University, Lund, Sweden

**Keywords:** Multiple sclerosis, depression, cognitive behavioural therapy, psychotherapists, content analysis

## Abstract

**Purpose:** To investigate how psychotherapists experience using individual, face-to-face cognitive behavioural therapy (CBT) aimed at alleviating depressive symptoms in persons with multiple sclerosis (MS).

**Method:** Semi-structured interviews with three psychotherapists were conducted after CBT with 12 participants with MS, and analysed using qualitative content analysis.

**Results:** Two main themes emerged: Trusting their expertise as psychotherapists whilst lacking MS-specific knowledge, and The process of exploring the participants’ readiness for CBT with modifications of content and delivery. The psychotherapists perceived it difficult to know whether a symptom was attributable to depression or to MS, and for some participants the CBT needed to be adapted to a more concrete content.

**Conclusions:** Psychotherapists may need more MS-specific knowledge and an insight into the individual’s functioning. The content of CBT in terms of concrete home assignments and behavioural activation needs to be individualised.

## Introduction

Depression is common in people with multiple sclerosis (MS), with a prevalence of approximately 30% which is higher compared to the prevalence in the general population (Marrie et al., 2015). The UK National Institute for Health and Care Excellence (NICE) guidelines recommend cognitive behavioural therapy (CBT) for treatment of depression in chronic physical health problems (NICE, 2009). Several studies have concluded that different modalities of CBT can be effective for alleviating depression in people with MS, such as group intervention (Forman & Lincoln, 2010; Larcombe & Wilson, 1984; Lincoln et al., 2011), individual face-to-face therapy (Mohr, Boudewyn, Goodkin, Bostrom, & Epstein, 2001), individual telephone contact (Mohr et al., 2005, 2000), or via the Internet (Cooper et al., 2011). Nevertheless, studies are needed on how CBT can be tailored for people with MS (Hind et al., 2014), since there are several disease-related aspects, such as fatigue and cognitive impairment, that need to be considered (Johansson et al., 2007; McAlpine & Compston, 2006). In Sweden, the National Board of Health and Welfare (2010) recommends CBT for the treatment of mild to moderate depression in the general population. However, it is not known how these recommendations apply for people with concurrent chronic conditions such as MS.

The current research group has performed a pilot feasibility study of individual face-to-face CBT for alleviating depressive symptoms in people with MS, as a preparation for a larger controlled study evaluating the effects. In line with the Medical Research Council’s recommendations for complex interventions (P. Craig et al., 2008), both quantitative and qualitative evaluations have been performed (Richards & Hallberg, 2015). In 2016, we reported quantitative results (Chruzander et al., 2016) and qualitative findings based on interviews with the people with MS who participated in CBT (Gottberg et al., 2016). We concluded that the depressive symptoms showed a tendency to decrease (Chruzander et al., 2016), and that from the perspective of people with MS, individual face-to-face CBT seems feasible for alleviating their depressive symptoms (Gottberg et al., 2016). However, preparing the participants for the time- and energy-demanding process of CBT, as well as increasing knowledge of MS among the psychotherapists, may facilitate participation. The perspectives of the psychotherapists delivering the CBT also need to be considered to gain broader knowledge of how CBT may need to be modified for people with MS. Thus, the aim of the present study was to investigate the psychotherapists’ experiences of using individual, face-to-face CBT aimed at alleviating depressive symptoms in people with MS.

## Material and methods

The design was qualitative, with semi-structured interviews analysed using latent or interpretive qualitative content analysis with an inductive approach (Downe-Wamboldt, 1992; Graneheim & Lundman, 2004) in order to describe the psychotherapists’ experiences of using CBT to alleviate depressive symptoms in people with MS. Since the 1950s, many researchers from a variety of research traditions have applied content analysis. Initially, content analysis was defined as ”the objective, systematic and quantitative description of the manifest content of communication” (Berelson, 1952). However, during the past two decades it has developed into a commonly used qualitative method within the hermeneutic tradition, with a focus on interpretations of latent content on meaning and experiences. In general, texts are the exclusive subjects of content analysis (Bos & Tarnai, 1999; Graneheim & Lundman, 2004). This study applies a hermeneutic perspective via a systematic analysis process to convey the meaning of texts, i.e. interpretive reading.

### Recruitment and participants

Aiming at including a strategic sample of 15 people with MS in the pilot feasibility study evaluating CBT for people with MS and depressive symptoms, it was considered necessary to recruit a total of three psychologists. The recruitment was performed using an advertisement at the website of the Swedish Association for Behaviour Therapy. The psychotherapists were informed both orally and in written form advising them of their right to withdraw at any time without the need for giving a reason. They were all licensed as psychotherapists, specialized in performing CBT, and private care providers. They were all women, and had a mean age of 55 years. They had worked as psychotherapists for an average of 9 years, but had either no or very limited prior experience of working with people with MS. The three psychotherapists each treated three or four people with MS. The Regional Ethical Review Board in Stockholm approved the study (2011/378-31/2).

### CBT intervention

The CBT intervention for each participant included 15–20 individual face-to-face sessions of 50 min each in duration, approximately one session per week (National Board of Health and Welfare, 2010) over an average duration of 25 weeks. At the sessions, the psychotherapists used Socratic questions, aimed at changing negative automatic thought processes (Barlow, 2014). The CBT included behavioural activation, such as agreed-upon tasks that the people with MS were to perform between sessions, cognitive interventions, and relapse prevention of depressive symptoms. The CBT sessions were free of charge for the people with MS and took place at the clinical practices of the psychotherapists. The people with MS were recruited from the Department of Neurology at Karolinska University Hospital Huddinge, Stockholm, Sweden. People with MS who, in conjunction with an outpatient visit or a telephone call to a nurse or a neurologist, were perceived as having depressive symptoms were screened for inclusion. Inclusion criteria were a definite and informed MS diagnosis and sub-threshold to moderate depressive symptoms [i.e. 11–19 points on the Beck Depression Inventory-II (Beck, Steer, Ball, & Ranieri, 1996)]. Exclusion criteria were an age below 18 years, other severe neurological or psychiatric disease, antidepressant medical treatment prescribed less than 3 months before inclusion and/or other ongoing psychological treatment. Twelve of the 15 included patients were women, the mean age at inclusion was 38 years, and 12 people had a mild MS severity [Expanded Disability Status Scale (Kurtzke, 1983)] assessed as 3.5 or lower. Of the 15 people with MS included, nine completed 15–20 sessions of face-to-face CBT, and six discontinued the intervention. Of those who did not fulfil the CBT, three had participated in two or more sessions of CBT.

### Data collection

The data collection was performed at scheduled appointments at the clinical practices of the psychotherapists, after the CBT intervention had ended.

The data were collected through one semi-structured face-to-face interview, with each of the three participating psychotherapists individually. To stimulate in-depth discussions and to obtain more substantial data, a duo interview with two of the psychotherapists was performed. At the duo interview, an observer interviewer was present to take notes of the discussion. The interviews were digitally recorded and transcribed verbatim. A semi-structured interview guide was used, which started with the question: “What is your experience of treating people with MS for alleviating their depressive symptoms with CBT?” This main question was followed up with additional questions to obtain a deeper understanding of what was beneficial and in what manner, of advantages or disadvantages, and of practical aspects regarding the delivery of CBT.

### Analysis

A qualitative content analysis (Downe-Wamboldt, 1992; Graneheim & Lundman, 2004) was conducted to describe the psychotherapists’ experiences, as well as the underlying meaning of those experiences. Thus, both manifest and latent content analysis was performed, according to the steps described in Table I. The two authors (KG and CY) analysed the text from two interviews each, and frequently discussed questions and uncertainties in Steps 1–4. A third author (GA) took part in this process, and reviewed difficulties that raised the need for discussion. The analysis was a reciprocal process from part to wholeness and back to part and wholeness in constant comparisons. At Steps 5 and 6, KG and CY conducted the analysis together to address the trustworthiness of the analysis, and the sub-themes and themes were refined at several meetings (Table 1).

**Table 1.** The content analysis process of the psychotherapists’ experiences of cognitive behavioural therapy (CBT) for people with multiple sclerosis and depressive symptoms.

Step 1. The transcribed interviews were read through in their entirety several times in order to get a sense of their content.
Step 2. The text was divided into meaning units, which consisted of the words and sentences that were related to each other through their content.
Step 3. The meaning units were condensed into shorter text that still contained the core of the meaning units and without interpretation.
Step 4. Each condensed meaning unit was labelled with codes, in a process of abstraction interpreting the underlying meaning.
Step 5. All codes were then compared with each other, and those with similar content were generated into sub-themes. The initial number of sub-themes was then reduced through a process of reading the codes, including the interpretation of the underlying meaning, and considering the whole context, thereby moving between the whole and the parts of the texts.
Step 6. The sub-themes were analysed according to their latent content, answering the question of *how* the psychotherapists experienced the CBT, and two themes were created based on the underlying meaning of the sub-themes.

## Results

Two themes emerged from the analysis, comprising a total of five sub-themes. The first theme was “Trusting their expertise as psychotherapists while lacking MS-specific knowledge”. This had two sub-themes: “Understanding the complexity of living with MS” and “Perceived lack of knowledge of MS giving rise to insecurity”. The second theme was “The process of exploring the participants’ readiness for CBT with modifications of content and delivery”. It consisted of three sub-themes: “Expectations as to the patients being more prepared for undergoing a demanding therapy”, “Motivated for CBT despite cancellations and problems with logistics”, and “Modifications to make the CBT more concrete”.

### Trusting their expertise as psychotherapists while lacking MS-specific knowledge

#### Understanding the complexity of living with MS

The psychotherapists’ understanding of the participants’ functioning developed gradually during the course of the individually oriented CBT. There was increasing awareness of how the participants tended to cover up their cognitive difficulties in social contexts and at work. The psychotherapists perceived that the participants did not face up to and accept their difficulties, but instead tried to hide them and achieve as much as before, especially at work. But the attempt to carry on as before led to the person’s being disappointed. The psychotherapists indicated that the depression appeared to have emerged in a chaotic situation where it was very difficult to balance work and leisure, and where the person’s total energy was expended on the attempt to maintain a functioning everyday life.

It depends on what’s given priority, you might say. Some of them put their work first, which is maybe natural if you want to keep your job. Yes, work’s important to them. But at the same time we’ve had a couple of them who’ve been afraid to say at work that they’ve got MS—they try to cover it up. They stay later because they can’t get the work done in the time it took before—being ill, they get tired more quickly. Covering things up like this must take quite a bit of energy. (Psychotherapist 2)

The psychotherapists perceived that some of the participants appeared not to have accepted the fact that they had been diagnosed with MS, for which reason they had no understanding of the impact of living with the disease. This was described in terms of these participants’ inability to see the connection between, on the one hand, their fatigue and cognitive difficulties, and, on the other, their need for support and the lightening of burdens in everyday life.

When I say “acceptance” I’m thinking more of facing the fact that you’re changed, that you don’t function as you used to, and of being able to talk about difficulties, so that you can say that you need this or that, or that you need changes at your place of work. It’s a question of not pretending that you don’t have these problems. I think that in two cases the depression was very much bound up with there being a small gap between the way things really were and the way the person thought things ought to be, given the circumstances. It’s very obvious there’s greater suffering when it’s like that. (Psychotherapist 3)

The psychotherapists perceived that either a worsening of the disease or the fear of such a worsening could cause deeper depression. They perceived the participants as suffering from mild to moderate depression, but felt that the primary problem was not always depression but might instead be anxiety, for instance. One of the psychotherapists reflected on the connection between anxiety and knowledge of what the disease implied. The psychotherapists regarded it as important to distinguish between depression and other problems of mental health that might come to light during the course of CBT and require primary treatment (anxiety being the most obvious example). The psychotherapists perceived a general alleviation of depression among the participants. In the case of those participants whose original depression was mild, however, it could be difficult to gauge the improvement, which chiefly took the form of a change in self-perception. It seemed that when the participants had achieved a better balance between work and leisure they acquired a confidence in the possibility of having a good life despite the troubles caused by MS. The psychotherapists reflected on there being a natural connection between behavioural activation and the improvement in quality of life, even though the essential purpose of CBT was to combat depression. It was seen as logical that the participants’ symptom management and general attitude were improved by the CBT. The psychotherapists felt that it was difficult, however, to draw far-reaching conclusions concerning the effect of CBT, since the group they treated was small and the variety of the results was large. In certain cases it was deemed that the depression was attributable to social issues and that CBT might not have been the best form of treatment.

#### Perceived lack of knowledge of MS giving rise to insecurity

Lacking prior knowledge of MS, the psychotherapists had learned most of what they knew about it from the participants. They expressed a certain worry concerning the possibility of new relapses of the disease among the participants and how such relapses should be handled within the framework of CBT. One psychotherapist had expected a great disparity in the different participants’ functioning, and felt a concern with regard to the possibility of a participant becoming gravely ill. The psychotherapists had expertise when it came to depression and CBT, but not when it came to MS, and they expressed a need for much greater specific knowledge of the disease. Although it had been useful to receive general information about MS before the CBT started, they felt the lack of more client- and treatment-oriented information. This knowledge would have been useful to acquire a better grasp of the specific depression issues and a better sense of what changes might be possible. There was a call for prior information about the degree of difficulty the particular participant had with cognition and fatigue (information preferably in the form of an assessment by a medical specialist), for the purpose of adapting the treatment to the individual. It was difficult for the psychotherapists to know whether a symptom such as fatigue was attributable to the depression or to the disease.

What was difficult, I’d say, was to distinguish the sort of tiredness that’s depressive, that’s a symptom of depression. But after a time I did feel that there was something specific about this sort of tiredness—specific to MS, I believe, and I thought that I could distinguish it, you might say. There was something I thought made it different from the depression I’d treated before. (Duo interviewees T2)

The psychotherapists also felt that they had insufficient knowledge concerning the various medicines that the patients were taking in connection with MS, and more specifically concerning possible side effects that were of significance with regard to depression. Furthermore, there was uncertainty as to what demands could be made of people with MS when it came to the activation of behaviour. What physical activities were feasible, for instance? It could be difficult to gauge the particular person’s limits.

It’s been more a question of how far I can urge the person on, how much I can expect of them. It certainly hasn’t been easy to know. With other patients this isn’t an issue—I can go by experience and as I get to know the person, I also get to know when they’re not really trying or don’t feel motivated enough. (Psychotherapist 1)

In the case of patients who are depressed but not otherwise ill, the psychotherapists know what demands can be made (however difficult it may sometimes be to activate a person with depression); in the case of MS patients, the psychotherapists felt like beginners.

### The process of exploring the participants’ readiness for CBT with modifications of content and delivery

#### Expectations as to the patients being more prepared for undergoing a demanding therapy

The psychotherapists felt that it could be an advantage not to start CBT too soon after a person had been diagnosed as having MS and started pharmacological treatment. It was difficult to conduct change-oriented therapy in the case of recently diagnosed patients who were going through a more or less critical psychological phase and were faced with an uncertain future. This factor could have affected the patients’ motivation with regard to participating in CBT. The psychotherapists perceived that certain people had a need to come to terms with the fact that they had been stricken with a chronic disease before they could turn to the task of surmounting obstacles in everyday life. It was important to make an overall judgement of the patient’s situation, and it was sometimes necessary to deal with other worries in everyday life before the treatment of depression started.

Sometimes the important intervention may be a matter of tackling the more fundamental needs, so to speak. (Duo interviewees Psychotherapist 1)

Generally, anyway, I don’t start CBT if the person’s got too many problems. You can’t conduct psychotherapy with a person who’s going through a crisis, for instance—the crisis has to be sorted out first. Either the patient mustn’t be with me or I have to bide my time. I mean, there can be an awful lot of things the person’s having to deal with—enormous problems at work and so on. If their lives aren’t in reasonably good order, patients can’t focus on the problem requiring therapy, but will instead just sit and talk so as to get things off their chests—and that’s not what we’re here for. (Duo interviewees Psychotherapist 2)

Since some of the participants did not have a clear-cut depression diagnosis, but did have other diagnoses as well as MS, the psychotherapists would have liked the assessment concerning depression to have been made by a psychologist or psychiatrist, as they were of the opinion that neurologists do not always have the requisite knowledge for making such an assessment. Not least among the points they made, was that a clear-cut diagnosis of depression is important in the context of studies (whose results may otherwise be misleading).

Yes, it should be a psychiatrist who makes the assessment about depression, or someone else with the right expertise. As I understood it, it was a neurologist that had made it, which is not a good idea at all. Unless it’s someone specializing in both fields—but that’s very unusual indeed. A neurologist remains a neurologist, not suited for assessing depression. (Psychotherapist 1)

In light of the fact that participation in CBT is not a passive process, the psychotherapists drew attention to the importance of preparing the participants in a study (as distinct from patients who have themselves applied for CBT and are highly motivated to take part) for what CBT involves in terms of time and commitment. Where CBT is offered (rather than sought), there may be less motivation and the participants may find the work involved too much for them. Furthermore, the psychotherapists considered that it would have been good if there had been a charge for the therapy, since payment can stimulate motivation. The fact that the treatment was free may have had something to do with the number of cancellations. Overall, the psychotherapists were nevertheless ready to receive patients with MS in the future.

#### Motivated for CBT despite cancellations and problems with logistics

The psychotherapists felt that the participants appreciated the therapy and could identify areas that needed working on. However, the number of late cancellations was striking. The patients were motivated and interested once they got to the sessions, but the “getting to” part could constitute an insurmountable obstacle; the person’s physical difficulties might be too great and the distance to be travelled too daunting.

I see them as having been motivated once they’d got here. Yes, I’d say they were interested and motivated—but the issue was perhaps rather the threshold they had to cross in order to get here in the first place. (Psychotherapist 1)

The psychotherapists found it difficult to handle the many cancellations. They felt that the participants identified themselves with their disease and spent a lot of time checking how they felt.

Patients have to be able to go by how they’re feeling and stay at home if the symptoms call for it …. I had two patients especially who rang and cancelled quite a few times. They appeared to be clearly identifying with their illness, checking every last aspect of it: ”How ill am I feeling today?” It was difficult—it is difficult—to know how to handle this as a psychotherapist. You’re in a position where it can be difficult to broach the subject in the right way. (Duo interviewees Psychotherapist 2)

Sometimes the psychotherapists felt ambivalent: while it was good that the participants knew their limits, it was not good that CBT could not be provided when the person did not show up to the therapy session. It was difficult to know whether the many cancellations were attributable to fatigue or to lack of motivation. One participant was put under stress by having sick children and having difficulty in sorting out the logistics with her husband, for which reason she broke off therapy despite a promising start. Another participant dropped out because she was undergoing other treatment at the same time and had long been participating in various therapies. The psychotherapists thought that the duration of the sessions was usually adequate, although sometimes a session had to be cut short because of a patient’s fatigue. They wondered, on the one hand, whether shorter and fewer sessions might not have been sufficient, and, on the other hand, whether, in light of the number of cancellations, a reduction in the number of sessions right from the start might not have made the participants more focused and more motivated.

#### Modifications to make the CBT more concrete

The therapy had to be adapted in the case of certain people who had such a poor executive capacity that they had trouble with abstraction and with incorporating the strategies they had acquired into their own lives. In the face of such trouble, the CBT should be on a more concrete level. However, it was difficult to generalize regarding the needs of people with MS as a group.

For my part, I found that I had to make the treatment a bit more concrete. I put it more, you might say, on the behavioural level, getting them going with physical and social activities. Yes, that’s where the emphasis was, I’d say, and the results have been good. (Psychotherapist 1)

The psychotherapists had been able to work in their usual way with some of the patients, while in the case of patients with cognitive difficulties they had had to take things more slowly. For some of the participants, CBT was a sluggish and laborious process, according to the psychotherapists. Some of the participants needed to focus on memory aids, acceptance of the MS diagnosis, facing the reality of their situation, and facing the fact that it was necessary to plan the day’s activities. Despite the difficulties and the need to adapt the CBT to give it a more concrete content, it was still possible to work towards the achievement of established goals. Although the main focus was on ordinary CBT against depression, much attention was also given to factors indirectly connected to depression, such as family situation, relationships, and communication. Although the therapy did not cover the issue of coming to terms with the MS diagnosis and mourning the decline in health, it fully covered the effects of the disease and the patients’ management of these effects in daily life. MS came up in the conversations, but was not the centre of attention. The psychotherapists indicated that if depression was accompanied by physical illness (such as MS) which affected the functioning of the brain, the question of whether the treatment needed to be modified became more complex. There was not the same complexity when it was a question of multiple mental diagnoses. Difficult decisions had to be made concerning the volume and intensity of the activation of behaviour. On the one hand, there was the need to activate; on the other, there was the issue of the participants’ fatigue.

Yes, it’s very different from how we need to work in the case of depression, where it’s a question of activation—I mean the activation of behaviour is an important part of the treatment of depression. But when MS fatigue comes into the picture it’s quite simply counter-productive to try to activate behaviour—it just wears the patient out. As I understand it, the patient needs to rest when the patient needs to rest, so to speak. But it isn’t like that when you’re using CBT against depression; it’s instead very much a question of going against the fatigue. (Psychotherapist 2)

The psychotherapists further made the point that the participants did their “homework” in different ways. On account of the problems these people had with regard to concentration, memory, and indeed simply getting started, the tasks to be performed at home were difficult and sometimes the initial level of ambition proved unrealistic and had to be lowered. Sometimes the tasks were not performed because of fatigue, but this was not necessarily MS related because it was encountered in the case of other patients too. The therapists pointed out that the work of bringing about change by means of CBT is founded on what is done (and how it is done) between the sessions, and that when CBT does not function it is often because the homework part is not performed. In light of this, the therapists expressed the wish that the continuance of the therapy should be conditional on the completion of the homework. Furthermore, the psychotherapists drew attention to the fact that it was difficult to push the participants with regard to identifying obstacles and overcoming them because an obstacle such as fatigue is formidable in the case of MS. On the other hand, it was found valuable to start from things that the participant liked.

## Discussion

To our knowledge, this is the first study to explore the experiences of psychotherapists using CBT for alleviating depression in people with MS. Two themes emerged from the interviews with the psychotherapists, describing their experiences of treating depression in people with MS through individual face-to-face CBT. The psychotherapists described that they were “trusting their expertise as psychotherapists while lacking MS-specific knowledge”, and they described their own process as “a process of exploring the participants’ readiness for CBT with modifications of content and delivery”.

The results of the present study showed that the psychotherapists saw improvements in mental well-being and decreased depressive symptoms of their CBT participants. Through treating people with MS, the therapists learned how depressive symptoms occurred in problems with acceptance and managing balance between demands in everyday life (i.e. at work) and capacity dependent on MS-related disability. This is in accordance with the results from our previous exploration of the experiences of face-to face CBT in people with MS, where the participants gained increased self-knowledge and a greater sense of well-being through CBT (Gottberg et al., 2016). Although CBT is a collaborative process (Dobson & Dobson, 2013), the psychotherapists described that they lacked knowledge of MS in general and particularly knowledge about the impact of MS on the individual patient that they treated. This lack of MS knowledge appeared to have hampered the CBT process, since the psychotherapists were uncertain of what demands they could put on the participants in terms of behavioural activation, a core element of CBT (Barlow, 2014). Fatigue was seen as both impeding behavioural activation and challenging to differentiate from depressive symptoms (Bakshi et al., 2000). Previous studies of CBT aiming at reducing the impact of fatigue among people with MS have shown that behavioural activation is feasible and effective (van Kessel et al., 2008), but the activities may need to be modified and individualized with regard to, for example, intensity and suitable time of day. This highlights the importance of MS-specific knowledge among psychotherapists, such as the daily fluctuations in functioning in people with MS (Ytterberg, Johansson, Andersson, Widén Holmqvist, & von Koch, 2008), as well as the need for a detailed referral with information on functioning of the individual person with MS and interdisciplinary collaboration.

The psychotherapists described that the CBT for some individuals had to be adapted to a more concrete level, focusing mostly on the behavioural component of CBT. This finding may imply that CBT might be more suitable for people with MS compared to other psychological therapies owing to its practical structure; however, for some individuals the cognitive component may need to be modified.

The psychotherapists also noted many late cancellations and described this as partly due to lack of motivation. In line with a person-centred approach, different modalities of CBT may be suitable for subgroups of people with MS. For example, CBT via the Internet (Cooper et al., 2011) may reduce the many late cancellations in some individuals, whereas for others needing more individualized therapy and human input (Hind et al., 2010), face-to-face CBT may be more appropriate.

It was evident that the psychotherapists wished that the people with MS had been more clearly diagnosed according to criteria for depression and that the participants should have had their other health needs taken care of before initiation of the CBT. However, in a clinical setting situated in neurological outpatient care, psychiatrists are often not available for screening or assessing people with MS for depression. Thus, screening measures of depressive symptoms suitable for the regular clinical setting need to be identified and used. The Swedish National Board of Health and Welfare (2010) also recommends CBT for mild to moderate depression, and in some cases, fluctuations in the severity of depression over time may occur (Ytterberg et al., 2008), for example from inclusion in a study or referral to the time of the first session of CBT. This may have been the case in the present study. A systematic review of the literature about CBT for depression in people with chronic neurological conditions showed that the severity of depression varied greatly among the studies, and therefore suggested more clearly defined inclusion criteria (Fernie, Kollmann, & Brown, 2015). In future studies on the effectiveness of CBT, a reassessment of depressive symptoms may be considered before CBT initiation in people with MS.

Some participants in the CBT were relatively newly diagnosed with MS, and the psychotherapists in the present study believed that their depressive symptoms were related to a crisis reaction, which was difficult to relate to in planning the individual CBT. As suggested earlier, future studies evaluating the effect of individual face-to-face CBT should consider not including people recently diagnosed with MS (Gottberg et al., 2016).

An evidence-based method such as CBT (National Board of Health and Welfare, 2010) in this study cannot always be directly implemented, because conditions such as MS may require adaptations, just as the results in this study have shown. Some authors argue that successful interventions are dependent on adaptations (B. Craig et al., 1987). On the other hand, programmes with high fidelity to the original programme model have had better outcomes than programmes with lower fidelity (Becker, Smith, Tanzman, Drake, & Tremblay, 2001; Keith, Hopp, Subramanian, Wiitala, & Lowery, 2010). Therefore, the effects of face-to-face CBT for alleviating depressive symptoms adapted for people with MS need to be evaluated in a larger study. In addition, our results showed that acceptance of the diagnosis and MS-related limitations in everyday life seemed to be important components in a psychological intervention for people with MS. Thus, other therapies such as acceptance and commitment therapy (Nordin & Rorsman, 2012) may also be suitable.

In this study, the perspective of psychotherapists delivering CBT for alleviating depressive symptoms in people with MS has been elucidated for the first time. A limitation of the study was that only three psychotherapists needed to be included owing to the small sample of people with MS. However, a duo interview was conducted, which contributed to more substantial data. Data from all interviews contributed to the themes and sub-themes. Two of the authors (KG and CY) frequently discussed the coding of meaning units and worked together in analysing the codes into sub-themes and themes, which is believed to have ensured the trustworthiness of the results.

## Conclusion

The findings imply that psychotherapists may need more MS-specific knowledge in general, as well as insights into the individual’s functioning, to alleviate depressive symptoms in people with MS. The content of CBT in terms of concrete home assignments and behavioural activation needs to be individualized. This knowledge will be valuable in the design and performance of a larger controlled study evaluating the effects of face-to-face CBT for alleviating depressive symptoms in people with MS.
